# Cationic fluorinated micelles for cell labeling and ^19^F-MR imaging

**DOI:** 10.1038/s41598-024-73511-8

**Published:** 2024-09-30

**Authors:** Natalia Jirát-Ziółkowska, Vyshakh Manayath Panakkal, Klára Jiráková, Dominik Havlíček, Ondřej Sedláček, Daniel Jirák

**Affiliations:** 1 Institute for Clinical and Experimental Medicine, Vídeňská 1958/9, Prague, 140 21 Czech Republic; 2 Institute of Biophysics and Informatics, First Faculty of Medicine, Charles University, Kateřinská 1660/32, Prague, 121 08 Czech Republic; 3 Department of Physical and Macromolecular Chemistry, Faculty of Science, Charles University, Hlavova 8, Prague, 128 00 Czech Republic; 4 Third Faculty of Medicine, Charles University, Ruská 87, Prague, 100 00 Czech Republic; 5 Faculty of Health Studies, Technical University of Liberec, Studentská 1402/2, Liberec, 461 17 Czech Republic

**Keywords:** Fluorinated micelles, Cell labeling, ^19^F magnetic resonance imaging, ^19^F magnetic resonance spectroscopy, Chemistry, Materials science

## Abstract

Magnetic resonance imaging (MRI) relies on appropriate contrast agents, especially for visualizing transplanted cells within host tissue. In recent years, compounds containing fluorine-19 have gained significant attention as MRI probe, particularly in dual ^1^H/^19^F-MR imaging. However, various factors affecting probe sensitivity, such as fluorine content and the equivalency of fluorine atoms, must be considered. In this study, we synthesized fluorinated micelles with adjustable surface positive charge density and investigated their physicochemical properties and MRI efficacy in phantoms and labeled cells. While the micelles exhibited clear signals in ^19^F-MR spectra and imaging, the concentrations required for MRI visualization of labeled cells were relatively high, adversely affecting cell viability. Despite their favourable physicochemical properties, achieving higher labeling rates without compromising cell viability during labeling remains a challenge for potential in vivo applications.

**Supplementary Information:**

The online version contains supplementary material available at 10.1038/s41598-024-73511-8.

## Introduction

Hydrogen Magnetic Resonance Imaging (^1^H-MRI) is the predominant MR technique in both clinical and preclinical applications. One notable application is the non-invasive monitoring of cell-based therapies. The use of MRI for cell tracking after transplantation has been extensively studied over several decades, encompassing both clinical^1,2^ and experimental studies^3–6^. However, the visualization of cells within the host tissue requires appropriate labeling using contrast agents (CAs). These CAs alter the relaxation time of the labeled cells, consequently enabling the differentiation from the surrounding tissue, inducing a signal change detectable on ^1^H-MRI^3,7–9^.

Nowadays mostly two basic types of CAs are used for ^1^H-MRI: *T*_1_ and *T*_2_. *T*_1_ agents, such as paramagnetic CAs based on gadolinium, are commonly utilized, leading to a hyperintense signal in ^1^H-MRI^10^. Conversely, *T*_2_ agents, like superparamagnetic iron oxide nanoparticles (NPs), induce a hypointense signal in ^1^H-MRI^11–14^. Several limitations are associated with those metal-based CAs. For instance, gadolinium, a commonly used metal-based contrast routinely applied in clinical examinations, may accumulate not only in pathological tissues but also in various organs such as the kidneys, potentially leading to considerable side effects^15,16^. Furthermore, iron oxide NPs may lack specificity, as hypointense spots observed on imaging can also arise from other pathologies, such as haemorrhage or trauma^13^. Given these limitations, current research is increasingly focused on non-metal CAs or probes, with particular attention on non-hydrogen nuclei, referred to as X nuclei (e.g., ^31^P, ^19^F, ^23^Na, ^39^K and ^17^O), which possess suitable properties for serving as MR probes^17–24^. Among these, stable natural monoisotopic fluorine ^19^F stands out as one of the most promising X-nuclei. The physiological concentration of fluorine in organisms is negligible, especially in soft tissues, making ^19^F-MR almost 100% specific, with minimal background noise. Another advantage of fluorine MR is its ease of quantification, as the detected signal is proportional to the number of ^19^F nuclei^21^. In addition, dual ^1^H/^19^F-MR imaging can be obtained using conventional scanners and radiofrequency (RF) coils with only minor hardware modifications, providing the ability to obtain an anatomical image (^1^H-MR) along with the fluorine signal (^19^F-MR). Fluorinated probes have proven to be valuable tools for various in vivo applications, including inflammation diagnosis^25^, measurement of protein metabolic activity^26^, cancer diagnosis^27^, and notably, cell visualization, where they have been successfully used to label various types of cells with minimal adverse effects^28–33^.

The general drawback of fluorine-based probe imaging may be the lower sensitivity, as some probes lack a sufficient amount of ^19^F nuclei for detection or possess chemically non-equivalent fluorine nuclei, resulting in multiple signals in ^19^F-MR spectra, and decreasing the MR visualization efficiency. Optimization of fluorinated probe structures is essential to ensure sufficient fluorine content and suitable relaxation times while maintaining hydrophilic properties. Increasing fluorine content, however, may lead to increased hydrophobicity^20,34–37^. Fluorinated probes are available in various forms, but the most common are perfluorocarbon emulsions stabilized by lipid surfactant^38^ and particles with perfluorocarbon core and polymer shell^39^. Perfluorocarbon-based probes have limitations due to high hydrophobicity and low solubility, rendering them less suitable for cell labeling and in vivo application. Hence, there is a search for new fluorine probes with enhanced properties. MRI probes based on self-assembled amphiphilic block copolymer NPs (micelles) comprise a fluorinated hydrophobic core shielded with a hydrophilic shell, providing stabilization, water-solubility, and biocompatibility. The size of the resulting NPs can be tailored within the range of 20–500 nm based on copolymer composition^40^.

To perform as an effective MR cell tracker, a probe must be internalized into cells, posing challenges in cell labeling with a sufficient probe amount for MR detection while preserving cell functionality and viability. Various approaches exist for cell labeling, with phagocytic cells like macrophages and dendritic cells being among the easily labeled. For non-phagocytic cells, indirect uptake *via* endocytosis may occur, although labeling efficiency depends on factors such as cell type, size of the cell and/or probe, and probe structure or chemical properties^41^. In some cases, there is a need to enhance the uptake in order to receive a sufficient MR signal. These methods may comprise electroporation, causing the change in the cell membrane permeabilization^42^, sonoporation, which refers to the formation of small pores in cell membranes by using ultrasound for the transfer of nucleic acid materials^43^, antibody-mediated specific labeling^44^, combining the probe with the poly-cationic transfection agent^13^, or hypotonic swelling^45^. The introduction of a positive charge to the NP surface represents a simple yet effective way to promote cellular association, as cationic NPs are electrostatically attracted to the negatively charged cellular membrane^46^.

In this study, we developed novel fluorinated micelles with varying positive charges in their shell and examined their sensitivity and overall suitability for application in ^19^F-MR imaging and cell labeling. The micelles have been synthesized by a one-pot method of polymerization-induced self-assembly, which allows straightforward synthesis and self-assembly of nanoparticles in a single step. The efficiency of these micelles for cell labeling was evaluated using fluorescence confocal microscopy and ^19^F-MR imaging, spectroscopy. Comprehensive cytotoxicity testing was performed to assess their potential as in vivo cell trackers. To demonstrate imaging properties, we performed a pilot in vivo experiment, where we subcutaneously administered the non-toxic fluorinated micelles to a healthy animal and monitored its sensitivity by ^19^F-MR.

## Methods

### Synthesis and characterization of cationic nanoparticles

#### Materials

If not stated otherwise, all chemicals were purchased from Sigma-Aldrich and were used without further purification. *N*-Hydroxyethyl acrylamide (HEAM) was filtered through a short pad of basic alumina before use to remove the inhibitor. *2*,*2’*-Azobis[2-(2-imidazolin-2-yl) propane]dichloride (VA-044) and *2*,*2’*-azobis(2-methylpropionamidine)dihydrochloride (VA-50) were purchased from TCI Europe. 2-(*n*-Butyltrithiocarbonate) propionic acid (BTPA)^47^ methyl 2-(*n*-butyltrithiocarbonate) propionate (MBTP)^48^ and *N*-(*222*-trifluoroethyl) acrylamide (TFEAM)^49^ were synthesized according to literature protocols. Water was deionized with a Millipore Milli-Q water purification system.

#### Synthesis of macroCTAs

Water-soluble trithiocarbonate chain end-containing macromolecular chain transfer agents (macroCTAs) were synthesized by reversible addition-fragmentation transfer (RAFT) (co)polymerization of neutral HEAM with positively charged (3-acrylamidopropyl) trimethylammonium chloride (APTMA) using either BTPA (for BA1-3 (co)polymers) or MBTP (for B1–4 series) CTAs at fixed monomer-to-CTA ratio of 100. Different [HEAM]_0_/[APTMA]_0_ ratios were used: 100/0 for homopolymers B1 and BA1, 90/10 for B2, 80/20 for B3, BA2, respectively and 0/100 for fully charged homopolymers B4 and BA3. Subsequent PISA extension of these macroCTAs resulted in M1 100/0, M2 90/10, M3 80/20, and fully charged M4 0/100 micelles.

In a typical experimental procedure, monomer(s) (17.39 mmol), CTA (BTPA or MBTP, 0.1739 mmol), and VA-501 (1.78 mg, 0.0434 mmol, [CTA]_0_/[VA-50]_0_ = 4:1) were dissolved in water-DMF (10 + 4 mL) mixture. This reaction mixture was purged with nitrogen gas and stirred in an aluminum heating block at 70 °C for 3 h. The reaction was quenched by exposure to air. The crude polymer solution was then dialyzed against deionized water overnight to remove the DMF and any other low-molar mass compounds. The macro-CTA was then recovered by freeze-drying, followed by characterization by SEC, NMR, and DLS.

#### Synthesis of fluorinated nanoparticles by PISA

Fluorinated block copolymer NPs were synthesized by RAFT-mediated dispersion polymerization-induced self-assembly (PISA) of TFEAM in distilled water. Typically, in the synthesis process of PHEAM_100_-*b*-[PTFEAM-*stat*-HEAM]_400_ (total solids content 6 wt%), TFEAM (528 mg, 3.45 mmol), HEMA (170 mg, 1.48 mmol), PHEAM-MBTP macro-CTA (145 mg, 12.3 µmol), VA-044 (1.32 mg, 4.11 µmol, [macroCTA]_0_/[VA-044]_0_ = 3:1) and 1,3,5-trioxane (5 mg) internal standard were dissolved in distilled water (13.06 mL), purged with nitrogen gas and stirred in an aluminum heating block at 50 °C for 5 h. The reaction was quenched by exposure to air, followed by ^1^H- and ^19^F-NMR analysis. Monomer conversion was determined by ^1^H-NMR spectroscopy of the reaction mixture upon dilution with CD_3_OD by comparing the residual vinyl peaks at 5.5–6.5 ppm with the signal of the internal standard. To calculate the ratio of both blocks, the NPs were freeze-dried, dissolved in CD_3_OD, and analyzed by ^1^H-NMR. The freeze-dried sample is then re-dissolved in phosphate-buffered saline (PBS, pH = 7.4) for further cellular experiments. For cellular fluorescent measurements, Rhodamine B octadecyl ester perchlorate was loaded into the micelles. The micelles (5 mL, 60 mg mL^− 1^) were labeled by incubating with the dye (5 mg) in a PBS solution, followed by continuous stirring for 48 h.

#### Characterization of polymers and nanoparticles

*Size exclusion chromatography* (SEC) was used to determine the molecular weights (*M*_w_ - weight-averaged molecular weight, *M*_n_ - number-averaged molecular weight) and dispersity (*Ð* = *M*_*w*_*M*_n_) of the polymers on an Agilent 1200 Infinity series system equipped with an Agilent 1200 Infinity Pump, a UV detector, and a RI detector. The separation was performed using a Novema MAX 100 Å 5 μm column thermostatted at 35 °C using an eluent of methanol with acetate buffer pH 5.5 (80:20 v/v) at an elution rate of 1 mL min^− 1^. Molar masses and dispersities were calculated against narrow-dispersity dextran standards.

*NMR spectra* were recorded on a Bruker Advance MSL 400 MHz NMR spectrometer at 25 °C in CD_3_OD, DMSO-d6, or a mixture of H_2_O/D_2_O (95/5 v/v). Unless otherwise stated, all ^19^F-NMR spectra were measured at *c*_*pol*_ = 30 mg mL^− 1^ using 20 µs pulse width, relaxation delay 8 s, acquisition time 1.5 s, and 64 scans, expressing all chemical shifts as ppm. The NMR spectra were processed using MestReNova 14.1 software, and the signal-to-noise ratios (SNR) were calculated using the built-in MestReNova function.

*Dynamic light scattering* (DLS) measurements were used to determine the hydrodynamic diameters of the polymers in distilled water on a ZEN3600 Zetasizer Nano-ZS zeta potential analyzer (Malvern Instruments, UK). The polymer samples (*c*_*pol*_ = 1 mg mL^− 1^) were filtered through a 0.45 μm PDFE syringe filter before measuring. The apparent Z-averaged hydrodynamic diameter of the particles, D_h_, was determined at a scattering angle of θ = 173°, and the DTS (Nano) program was used to evaluate the data.

The *Zeta potential* of NPs was measured by Zetasizer Nano-ZS. Each dispersion containing a polymer at a concentration of 1 mg mL^− 1^ was carefully filtered using 0.45 μm PDFE syringe filters into disposable zeta potential cuvettes. The measurements were performed in triplicates. To evaluate the stability, NPs were monitored by DLS again after 6 months.

### Cell labeling and viability

Accurate cell labeling is essential for effective cell visualization *via*^19^F-MR. In this study, we performed in vitro experiments using the grade IV prostatic adenocarcinoma (PC-3) cell line. Cells were cultured in Dulbecco’s Modified Eagle’s Medium/Nutrient Mixture F-12 Ham DMEM : F12 medium (Gibco™, Thermo Fisher Scientific, USA), supplemented with 10% fetal bovine serum (Gibco™, Thermo Fisher Scientific, USA) and 1% Penicillin/Streptomycin (Biosera, France) and incubated under standard conditions (T = 37 °C; 5% CO_2_). Initially, PC-3 cells were seeded in a 96-well plate at a density of 0.05 × 10^6^ cells per well and incubated in medium for 24 h. Subsequently, cells were treated with various concentrations of micelles (*c*_*pol*_ = 10, 15, and 20 mg mL^–1^) with different surface charges (M1 – M4) for 24 and 48 h in the cell cultivation medium.

Viability of labeled cells was assessed using the Alamar blue assay (Sigma Aldrich, St. Louis, MO, USA). Prior to spectrophotometric analysis, cells were washed three times with PBS (Gibco™, Thermo Fisher Scientific, USA). Then, a 10% Alamar blue solution was added, and cells were incubated under standard conditions for 4 h. Finally, absorbance was measured at wavelengths of 570 and 600 nm using a Tecan Infinite 200 PRO reader (Tecan Group Ltd., Switzerland), and the signal from treated cells was compared with positive and negative controls. Each experiment was performed in triplicate and repeated three times. To assess the fluorine MR signal in vitro, PC-3 cells were incubated in a 6-well plate (0.4 × 10^6^ cells per well) and cultivated as described above. The cells were then labeled for 24 h with two concentrations (*c*_*pol*_ = 10 and 20 mg mL^–1^) of different micelles types (M1 – M3; excluding M4 due to high cytotoxicity). For additional evaluation of M2 NPs signal on 7 T scanner, 4T1 cells were labelled using the same methodology (24 h incubation, *c*_*pol*_ = 20 mg mL^–1^).

### MR spectroscopy and imaging

MR in vitro measurements were conducted on a 4.7 T Bruker Biospec 47/20 MR scanner (Bruker BioSpin MRI GmbH, Ettlingen, Germany). MR characterization of the polymer and labeled cell dispersion was performed using a highly sensitive small custom dual ^1^H/^19^F RF solenoid coil (diameter = 8 mm, length = 8 mm, 4 turns) specifically designed for a small sample volume (*V* = 0.5 mL) to ensure high-quality MR signal quantification. For further measurements, especially relevant for in vivo applications, a dual ^1^H/^19^F RF surface coil designed for small laboratory animals was used. These coils were designed to facilitate on-machine tuning and matching at ^19^F and ^1^H Larmor frequencies (at 4.7 T 188 and 200 MHz, respectively).

Initially, micelles with various surface charges (M1 – M4, *c*_*pol*_ = 10 mg mL^− 1^) were placed in cell cultivation medium (*V* = 0.4 mL, 500 µL Eppendorf tube) and measured as phantoms. Two MR modalities were employed: ^1^H-MRI for localization (RARE sequence; repetition time TR = 3300 ms, echo time TE = 12 ms, field of view FOV = 4 × 4 cm, scan time ST = 1 min), and ^19^F-MRS for determining the fluorine signal (single pulse sequence; TR = 200 ms, ST = 33 min, bandwidth BW = 200 ppm) using a dual ^1^H/^19^F solenoid coil.

Following three washes with PBS, the labeled cells were collected for MR measurement as a cell suspension in fresh cultivation medium (*V* = 0.4 mL). The medium used during labeling served as a ^19^F-MRS signal reference. Labeled cells were measured using a dual ^1^H/^19^F surface coil for ^1^H-MRI and ^19^F-MRS (following the same protocol as for micelles alone) and ^19^F-MRSI (chemical shift imaging CSI; TR = 800 ms, FOV = 4 × 4 cm, slice thickness = 1.5 cm) with a scan time of 1 hour for micelles with medium alone and 26 min–8 h for labeled PC-3 cells.

Given the promising results of the M2 probe, we performed ^19^F-MR experiments on a 7 T Bruker BioSpec 70/30 using commercial available ^1^H/^19^F surface coil (Bruker BioSpin MRI GmbH, Ettlingen, Germany) as a proof-of-concept study. Initially, various micelles (M2, M1, M4; *V* = 200 µL, c_pol_ = 60 mg mL^− 1^) were measured to adjust the parameters using the same modalities as in the previous part and ^19^F-MRSI (CSI; TR = 200 ms, FOV = 4 × 4 cm, slice thickness = 2.0 cm) with a scan time of 3 min. The M2 NPs (V = 200 µL, c_pol_ = 20 mg mL^− 1^) were used to label 4T1 cells, which were then measured using ^19^F-MRS (single pulse sequence; TR = 200 ms, ST = 2 min, bandwidth BW = 180 ppm). Finally, we conducted a proof-of-principle in vivo MR, where the non-toxic nanoparticles with the lowest charge (M2; V = 200 µL, c_pol_ = 60 mg mL^− 1^) were injected subcutaneously into a hind leg of a healthy mouse and measured right after the injection (Day 0) and after 1 and 6 days (Day 1 and Day 6). On the opposite side, we placed a reference tube containing the same amount of nanoparticles.

Measurements were performed using ^1^H-MRI for localization (RARE sequence, TR/TE = 800/25, ST = 1 min, FOV = 4 × 4 cm, axial plane), non-localized ^19^F-MRS (TR = 500 ms, ST = 30 s), and ^19^F-CSI (TR = 200 ms, TE = 1.25 ms, ST = 20 mm, Scan time = 6 min, Matrix = 64 × 64). All measurements were obtained using a dual ^1^H/^19^F surface coil.

MR image analysis and following quantification were performed using the ImageJ 1.48 (National Institutes of Health, Bethesda, USA). Processing and quantification of MR spectroscopy were carried out using a custom MATLAB script (https://mathworks.com, Matlab R2021b, The MathWorks, Inc., USA).

### Confocal microscopy

Confocal microscopy was performed to validate the uptake of probes with different charges. PC-3 cells were incubated for 24 h using M1 and M2 micelles in various concentrations (*c*_*pol*_ = 10, 15, and 20 mg mL^–1^) and labeled with rhodamine fluorescent tags. Afterwards, the cells were washed three times with PBS and fixed (4% paraformaldehyde, 0.1 M PBS for 15 min), then washed again three times with PBS and embedded in Vectashield/DAPI (Vector Laboratories, Newark, CA USA) and analysed using an Inverted fluorescence confocal spinning disk microscope Olympus SpinSR10.

## Results and discussion

**Scheme 1:**
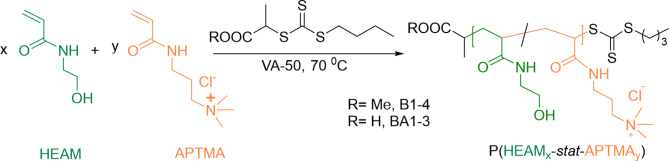
Synthesis of macromolecular chain transfer agents (macro-CTAs) with variable positive charge density.

The fluorinated cationic copolymer NPs were synthesized by RAFT-mediated radical PISA in water, where the cationic macroCTA was extended by *N*-(2,2,2-trifluoroethyl) acrylamide (TFEAM) forming the hydrophobic fluorinated block within the micellar core (Scheme 1). The macroCTA then becomes a hydrophilic shell that comes into contact with the biological environment. Therefore, careful design and introduction of charged units into the macroCTA are of crucial importance in this study. In our previous work^50^, we used poly(ethylene glycol)(PEG)-based macroCTA leading to a PEG micelle shell, which, unfortunately, does not enable the facile introduction of positive charges. Therefore, we synthesized novel macroCTAs based on water-soluble statistical copolymers of hydrophilic HEAM with positively charged APTMA, where the HEAM/APTMA ratio varied (100/0–0/100) to adjust the content of cationic groups. The copolymerization was performed by RAFT method in a water-DMF mixture using two different trithiocarbonate CTAs − 2-(*n*-butyltrithiocarbonate) propionic acid (BTPA, for polymers BA1, 3, and 4) and methyl 2-(*n*-butyltrithiocarbonate) propionate (MBTP, for polymers B1–4). The frequently used BTPA CTA provides polymers with carboxylic acid chain ends negatively charged at physiological pH and thus will partly reduce the micelle’s overall positive charge. On the other hand, methyl ester containing MBTP-based polymers will remain neutral, and only charges will arise from the presence of cationic APTMA units. The degree of polymerization of macroCTAs was kept constant at 100.

Polymers with different content of cationic APTMA were synthesized as hydrophilic shell-forming blocks, ranging from fully cationic PAPTMA homopolymers B4 and BA3 to polymers with significantly lower cationic charge content (B2, B3, and BA2 containing 10, respectively 20% of APTMA units) to improve their cytocompatibility. Finally, copolymers with no positive charge B1 and BA1 were synthesized as controls. The polymers were characterized by size-exclusion chromatography (SEC) and ^1^H-NMR (Figure S1), as well as ^1^-^13^ C HSQC-NMR, to resolve the overlapping peaks. All measurements confirmed the copolymer composition to be close to the target values (Table S1).

**Scheme 2:**
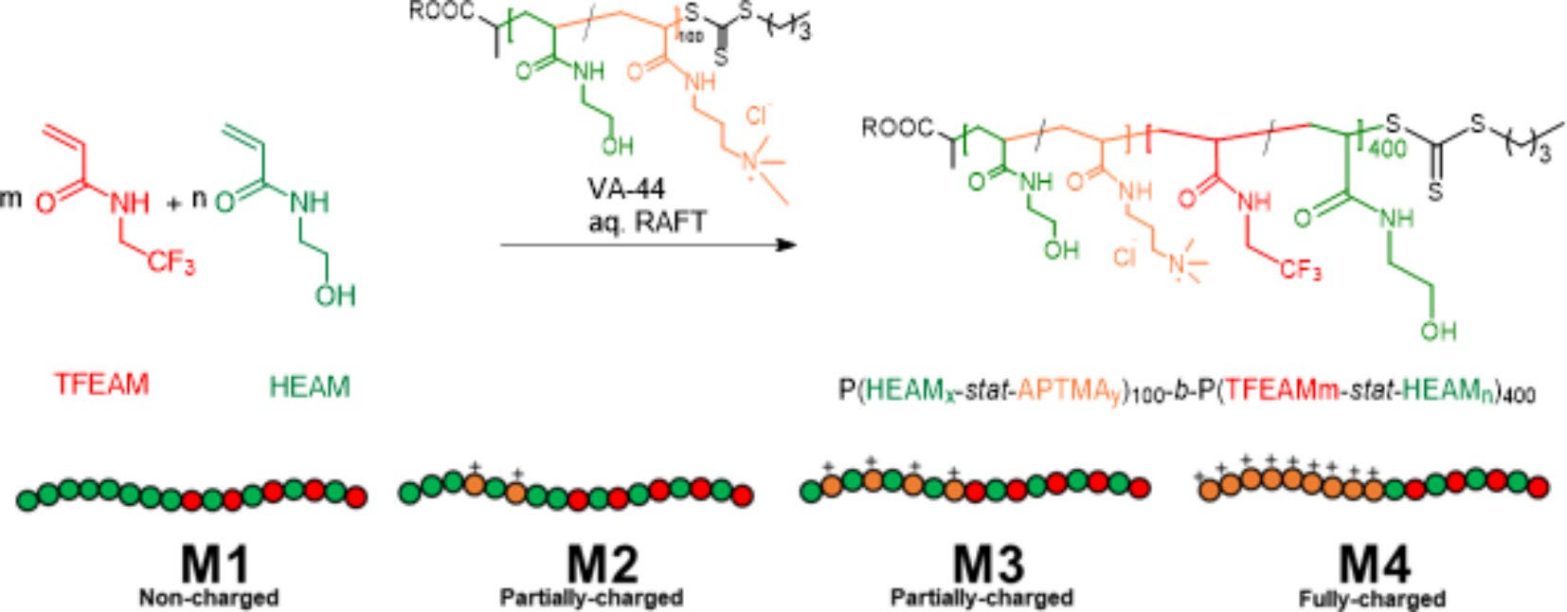
Synthesis of fluorinated block copolymer nanoparticles with controlled shell charge density.

The synthesized copolymers B1–4, respectively BA1–3, were then used as macro-CTAs for the synthesis of fluorinated block copolymer NPs M1–4, respectively MA1–3, by aqueous dispersion PISA. The macroCTA hydrophilic block was chain-extended by PTFEAM hydrophobic block to gain amphiphilicity. Particularly, TFEAM was used as a core-forming monomer due to its water-solubility in a monomeric state, while turning insoluble upon reaching a certain degree of polymerization (Scheme 2). This leads to in situ self-assembly of NPs during polymerization, providing the first block (macroCTA-based) remains water-soluble to stabilize the micelles. In all cases, the degree of polymerization of the fluorine-containing block was 400.

To improve the ^19^F-MR relaxation times of the fluorinated core, a small portion of HEAM (30 mol %) was added to the PISA reaction mixture in analogy to our previous findings^50^. The fluorinated core-forming block then consists of a statistical copolymer of P(FEAM-stat-HEAM), with the overall block copolymer structure being P(HEAM-*stat*-APTMA)_100_-*block*-P(TFEAM-*stat*-HEAM)_400_. The copolymer composition was confirmed by ^1^H-NMR; however, the attempts to perform SEC analysis of charged micelles failed due to the simultaneous presence of positive charges and fluorinated segments in copolymers, which led to strong interactions with the SEC column.

**Fig. 1:**
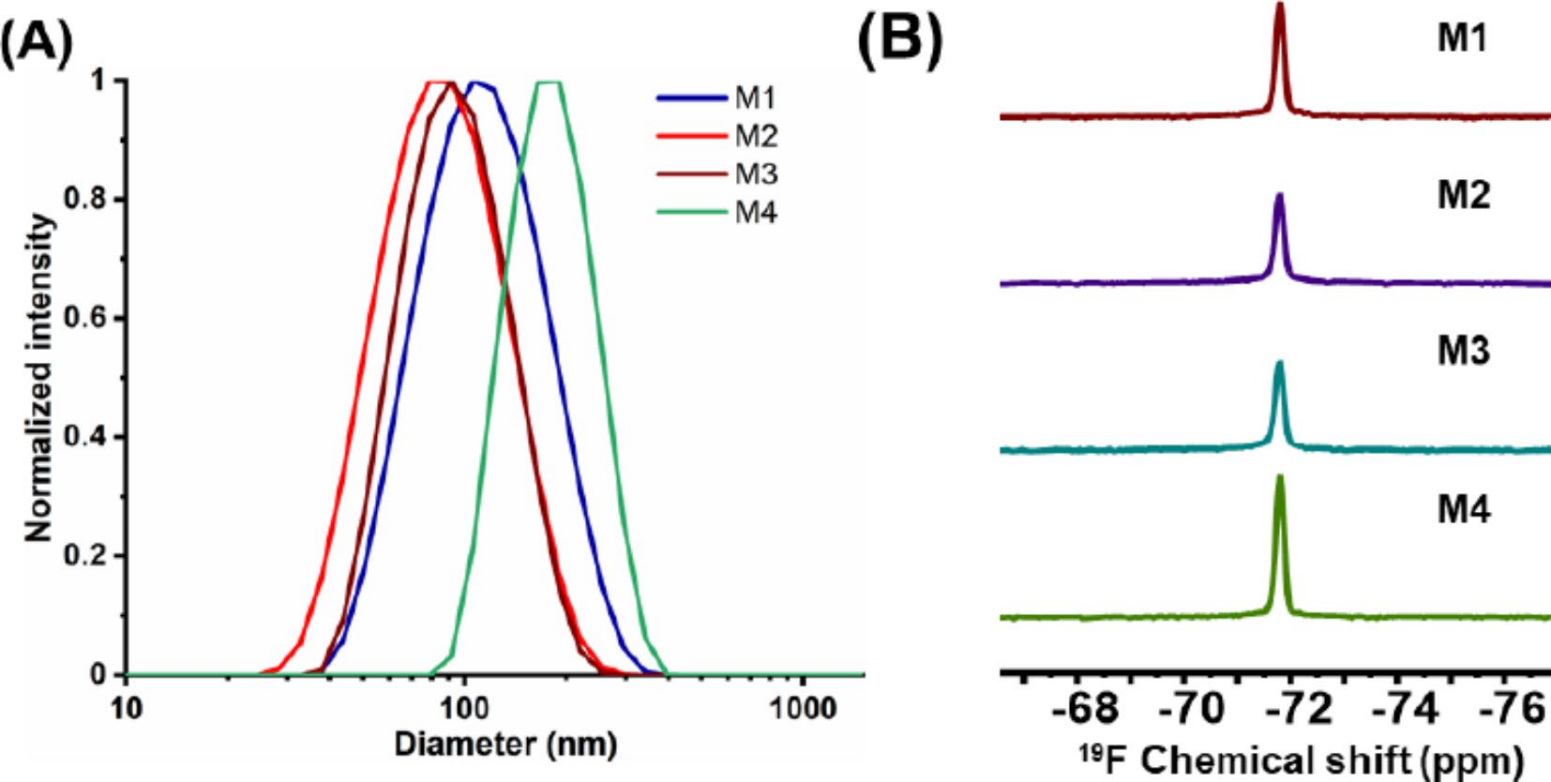
Physicochemical properties of fluorinated block copolymer nanoparticles M1 – M4 with controlled shell charge density. (A) Hydrodynamic size distribution in water measured by DLS (*c*_*pol*_ = 1 mg mL^− 1^). (B) ^19^F-NMR (400 MHz) spectra of nanoparticles in PBS (*c*_*pol*_ = 30 mg mL^− 1^).

On the other hand, successful chain extension was confirmed by the formation of NPs during polymerization. The hydrodynamic size of the NPs was measured by dynamic light scattering in the range of 61–148 nm, with the largest size belonging to NPs with fully charged shells (M4 and MA3, Table 1; Fig. 1). Furthermore, the electrophoretic zeta potential of micelles was determined as a measure of NPs charge density. Interestingly, this value was strongly dependent on the structure of the CTA-based chain end group. In the case of BTPA-based polymers, the single carboxylic acid group at the polymer chain end becomes negatively charged at physiological conditions, and the merely single negative charge influences the overall surface charge of the micelle. This is most apparent in the case of otherwise neutral PHEAM-shell NPs, which show a negative zeta potential of -15.2 mV for BTPA-based polymer MA1, while the analogous MBTP-based NPs M1 show a nearly neutral zeta potential of 0.13 mV. The zeta potential increases to positive values with increasing content of cationic APTMA comonomer. Based on these measurements, MBTP-based NPs M1 – M4 were selected for further investigations. The physicochemical properties of fluorinated block copolymer nanoparticles M1 – M4 are presented in Fig. 1. The long-term nanoparticle stability was confirmed by DLS performed on M2 micelles six months after synthesis (Figure S2). The stability based on MR signal is supported by long-term MR imaging of cells labeled with micelles, measured in a cell cultivation medium suspension. Additionally, no sifnificant difference was observed in the ^19^F-MR signal of M4 micelles (*c*_*pol*_ = 60 mg mL^− 1^) incubated for 24-hours with 20% FBS compared to M4 NPs alone (data not shown).

**Table 1:** Properties of fluorinated block copolymer nanoparticles P(HEAM-*stat*-APTMA)-*block*-P(TFEAM-*stat*-HEAM) with controlled shell charge density.

Polymer	End-group^1^	F_APTMA_ (%)^2^	D_h_ (nm)^3^	ζ-potential (mV)^4^	^19^F-NMR SNR*^5^
M1	-COOMe	0	101	0.1	157.3
M2	-COOMe	10	77	17.0	118.0
M3	-COOMe	20	87	26.8	121.4
M4	-COOMe	100	148	36.6	189.5
MA1	-COO^−^	0	64	− 15.2	117.8
MA2	-COO^−^	20	61	40.2	98.9
MA3	-COO^−^	100	142	54.2	132.3

^1^Chain end group at physiological pH. ^2^APTMA content in the hydrophilic block. ^3^Hydrodynamic diameter of nanoparticles determined by DLS in water (*c*_*pol*_ = 1 mg mL^− 1^). ^4^Determined by Zetasizer Nano-ZS in water (*c*_*pol*_ = 1 mg mL^− 1^). ^5^Fluorine NMR (400 MHz) signal-to-noise ratios in PBS (*c*_*pol*_ = 30 mg mL^− 1^).

For successful in vivo visualization of labeled cells, it is crucial to ensure a sufficient amount of probes in cells, allowing for effective tracking in MR. Importantly, this should not compromise cell viability, cellular processes, and functionality. Among the properties that affect NP internalization are size, shape, material, hydrophilic properties, and surface charge^51^. The internalization process in non-phagocytic cells is dependent on endocytosis, which precedes the interaction of nanoparticles with cells.

**Fig. 2:**
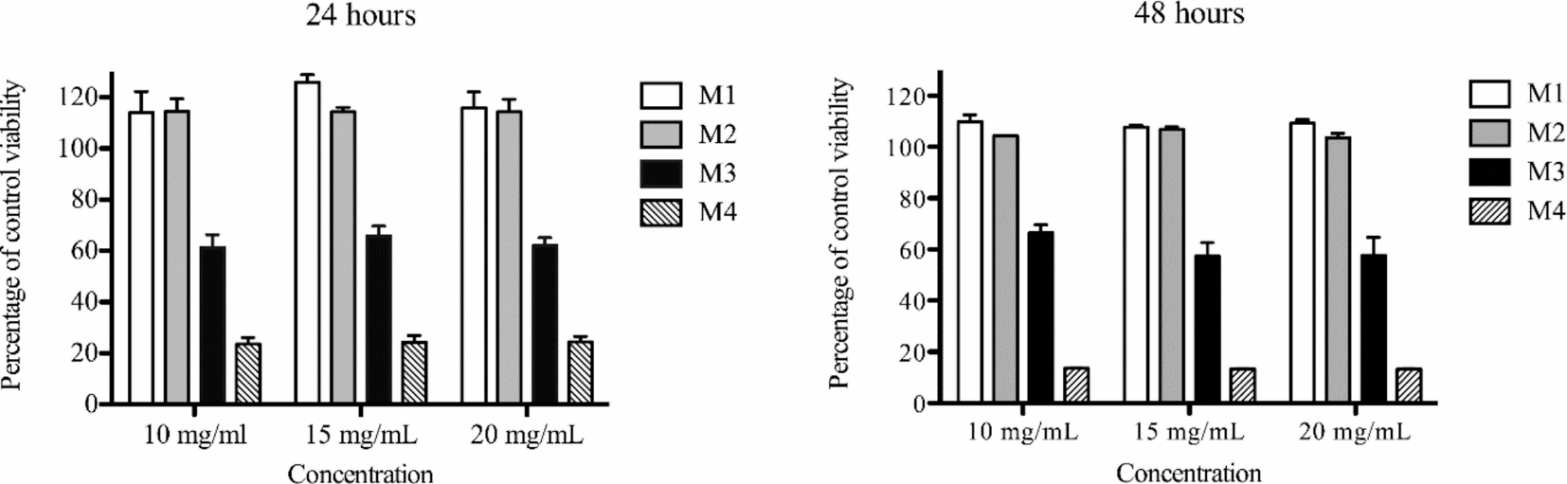
Impact on cell viability following exposure to fluorinated block copolymer NPs M1 – M4. Absorbance values were normalized, with the control cells set at 100%. Treated cells underwent labeling for 24 and 48 h using fluorinated particles with varying surface charge content (M1: non-charged, M2: 10%, M3: 20%, and M4: 100%) at concentrations of 10, 15, and 20 mg mL^− 1^. The data represent the mean of three independent experiments ± standard error of the mean (SEM).

We conducted cytotoxicity tests on various types of fluorinated cationic copolymer NPs at different concentrations for 24 and 48 h (Fig. 2). The viability of cells incubated with M1 and M2 nanoparticles (no charge and 10% charge, respectively) was comparable to control cells and was not affected by increasing concentration of polymer or by longer incubation time. The viability assay clearly shows that the fully charged micelles (M4) are highly toxic for cells even in low concentrations and short incubation period. Consequently, we excluded these nanoparticles from further MR experiments. In addition, the micelles with 20% positive charge (M3) caused a considerable decrease in the viability of exposed cells, where the viability decreased to approximately 60% of the control. This measurement corresponds with the observation of abnormal cellular morphology especially in cells incubated with the M3 nanoparticles (compared to cells labeled with M1 and M2).

In the literature, a positive surface charge has been demonstrated to enhance the cellular uptake of various nanoparticles, such as selenium nanoparticles^52^, polymeric particles in different cell lines^46^, and ZnO nanoparticles^53^. Unfortunately, cellular uptake of NPs with a positive charge is also associated with increased cytotoxicity, as shown in some studies. For instance, positively charged Au nanoparticles displayed toxicity and induced mitochondria stress at lower concentrations compared to their non-charged nanoparticles in the human keratinocyte cell line^54^. Similarly, the polycationic transfection agents like Lipofecatime, Poly-L-Lysine, or protamine sulphate, used to enhance cell labeling^55^, have been reported to exert toxic effects on cells^56^. The potential cause of this toxicity may be that cationic NPs cause more pronounced disruptions in plasma-membrane integrity, stronger mitochondrial and lysosomal damage, and a higher number of autophagosomes than anionic NPs^57^. It is evident that the charge of probes plays a crucial role in their cytotoxicity, but this can be affected by factors such as the amount, type or density of positively charged groups^57–59^. These findings correspond with our results, where the viability of cells labeled with M1 micelles is comparable to that of the control cells.

**Fig. 3:**
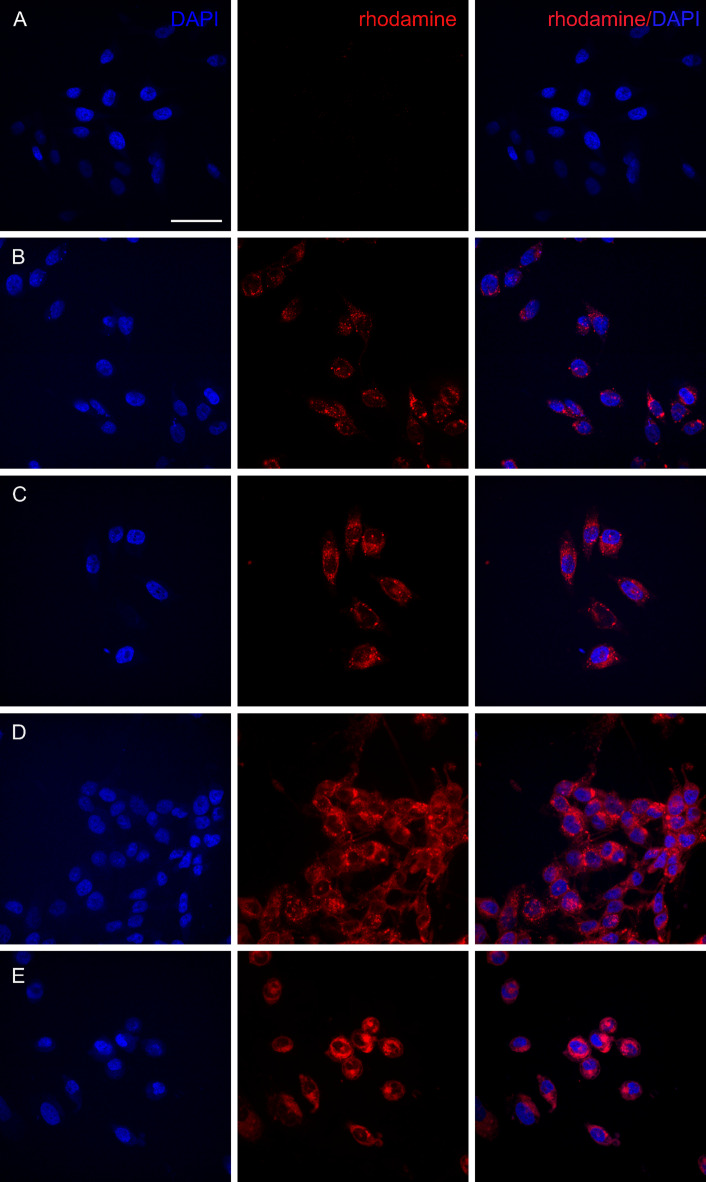
PC-3 cells labeled with Rhodamine-tagged micelles (red) and DAPI (blue). (A) Control cells. (B) Cells labeled with M2 at 10 mg mL^− 1^, and (C) 20 mg mL^− 1^. (D) Cells labeled with M3 at 10 mg mL^− 1^, and (E) 20 mg mL^− 1^. The scale bar represents 50 μm.

To confirm the internalization of micelles, confocal microscopy was conducted using micelles tagged with rhodamine B (Fig. 3). There was slight increase in cellular uptake in cells labeled with probe with higher positive charge and higher labeling concentration. However, a noticeable change in cell morphology was observed when comparing cells labeled with M2 and M3 micelles, In M3-labeled cells, there were significantly fewer cells on the slide, often appearing in clusters, and a prevalent observation of rounded cell morphology compared to cells labeled with M2 micelles. These findings align with the viability tests, indicating a more pronounced impact on viability in M3-treated cells.

**Fig. 4:**
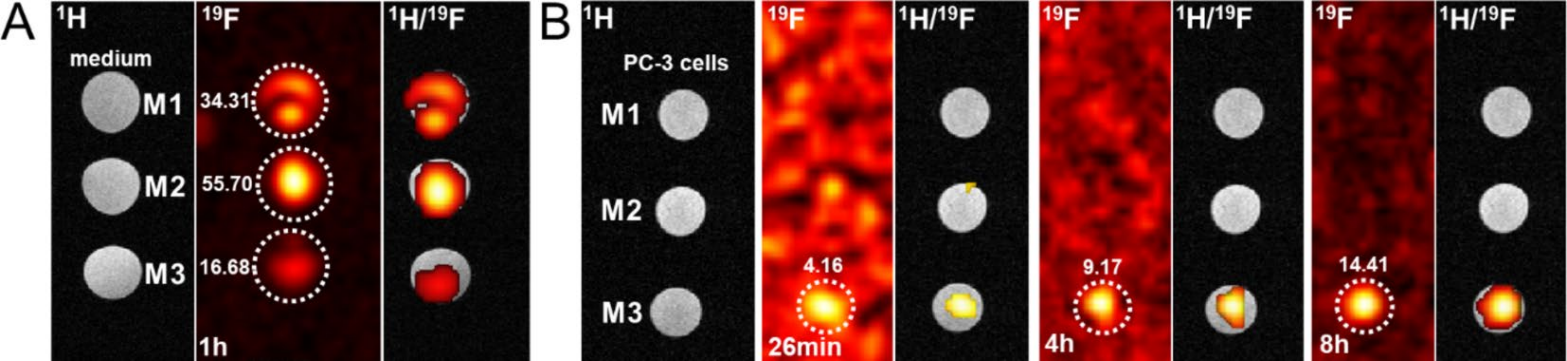
^19^F-MR results of different micelles. (A) ^1^H/^19^F-MRI/MRSI of M1 – M3 micelles in cell culture medium (*c*_*pol*_ = 20 mg mL^− 1^), scan time = 1 hour. (B) ^1^H/^19^F-MRI/MRSI of M1 – M3 micelles in labeled cells (*c*_*pol*_ = 20 mg mL^− 1^), scan time = 26 min, 4 h and 8 h. All measurements were obtained from a surface coil and simultaneously for every probe set. The overlaid ^1^H/^19^F-MRI is presented with the fluorine signal artificially marked in red.

In our investigation, we initially observed insufficient MR signal from cells labeled with uncharged ^19^F-containing micelles (Fig. 4A). To address this limitation, we decided to explore micelles with modified surface charges. As previously discussed, NPs with positive charges are expected to exhibit greater affinity for negatively charged plasma membranes, facilitating enhanced internalization compared to anionic or neutral nanoparticles^57^. The versatility of fluorinated polymers allows for modification and functionalization according to specific requirements. Hence, we chose to label cells with micelles carrying variable positive charges and investigated their impact on both cell viability and internalization efficiency.

The potential of cationic NPs as ^19^F-MRI tracers was investigated *in vitro.* Initial pilot measurements of M1, M3, and M4 micelles, resulted in a ^19^F-MRS signals for M3 and M4 in both phantoms (*c*_*pol*_ = 10 mg mL^− 1^) and labeled PC-3 cells (*c*_*pol*_ = 20 mg mL^− 1^, **Figure S3A**) when measured with a solenoid coil. Subsequently, PC-3 cells labeled with M3 and M4 micelles resulted in high ^19^F-MRSI SNR. Particularly, partially-charged M3 (*c*_*pol*_ = 10/20 mg mL^− 1^, SNR = 18.55/3.85) and charged M4 (*c*_*pol*_ = 10/20 mg mL^− 1^, SNR = 19.01/3.96) demonstrated significant SNR when measured using a surface coil (**Figure S3B**).

Further, we proceeded with lower-charged M2 micelles due to the observed high cytotoxicity of M4. Adequate uptake was detected with partially-charged M3 micelles. Analysis of M1 – M3 probes in cell culture medium extracted from labeled cells and measured using a surface coil resulted in ^19^F-MRSI signal for all charge modifications (SNR = 34.31, 55.70, 16.68, respectively; see Fig. 4A). M3 micelles at a higher concentration of 20 mg mL^− 1^ exhibited the most favorable outcome in labeled PC-3 cells, as visualized using ^19^F-MRSI (SNR = 4.16–14.41, ST = 26 min–8 h; Fig. 4B). Presented images were measured using a surface coil, which is more relevant for further in vivo experiments.

During the initial phases, we noted that high-SNR MR signals obtained from the medium might suppress signal from cells when measured simultaneously. Consequently, we decided to measure the medium with micelles and labeled cells separately. Results indicate that sufficient ^19^F-MRSI SNR can be achieved with relatively short acquisition times and low micelles concentrations. As expected, charged micelles resulted in higher cell penetration compared to uncharged micelles, with the best outcomes observed with micelles possessing 20% charge (M3). With the charged micelles we increased the labelling efficiency, however on the cost of higher cytotoxicity. The in vitro results show limitations of our 19-F tracer, to overcome these limitations, it is needed to align achieving higher signal at ^19^F-MR and less toxicity effect on labelled cells. Additional modifications of probe charge, considering viability requirements and higher amount of fluor content in the tracer to increase the ^19^F-MR sensitivity, will be explored in subsequent experiments.

Moreover, the micelles alone could serve as effective cell trackers in in vivo cell transplantations. The extensive imaging possibilities should be examined in optical imaging, as the probe was easily loaded with the Rhodamine B fluorescent dye. Fluorescent imaging can complement MR cell tracking, offering increased sensitivity, especially in short-time experiments both in vitro and in vivo. Furthermore, the attached dye could serve as a carrier for specific drugs. Theranostic approaches combining these modalities have been described in the literature^43,60,61^. This strategy may lead to real-time imaging of labeled cells using emerging imaging technologies^62^. The sensitivity of MR methods is dependent on the magnetic field strength. Given that our MR measurements were conducted on a 4.7 T MR system, which is closer to the magnetic fields used in clinical practice, the sensitivity is comparatively lower than that achievable with ultra-high magnetic fields. This difference in sensitivity may contribute to the obtained signal falling below its detection threshold, thereby explaining why cells with an internalized probe, as observed in fluorescence microscopy, did not produce a detectable signal in MR scans. In future studies, we aim to evaluate the probe’s sensitivity at a magnetic field of 7 T, to overcome these limitations.

To demonstrate the potential of fluorinated micelles at higher magnetic fields, we conducted a proof-of-concept in vitro and in vivo trial. Analysis of M2, M3 and M4 NPs resulted in high SNRs ranging from 7.07 to 54.36 on ^19^F-CSI in a short acquisition of 3 min (**Figure S4A**). 4T1 cells labeled with M2 micelles showed a ^19^F-MRS signal (**Figure S4B**). Subcutaneously injected M2 micelles produced a clearly detected MR signal, along with a phantom containing the same M2 probe concentration (**Figure S4C**). The MR signal from the micelles originated only from the injection region and the phantom and was separated by a large chemical shift from the signal of isoflurane anesthetic.

## Conclusion

In conclusion, we have successfully synthesized self-assembled amphiphilic block copolymer nanoparticles (micelles) with a fluorinated hydrophobic core designed for use in ^19^F-MRI, coupled with a controlled shell charge for cell labeling. These micelles exhibit water solubility and have a hydrodynamic diameter ranging from 61 to 148 nm. Importantly, all synthesised micelles demonstrate a clear single peak in ^19^F-MR spectra and imaging. Furthermore, our results indicate successful internalization of the micelles in cells, with a stronger positive charge correlating with enhanced MRI visualisation. However, this increase in positive charge also corresponds to higher cytotoxicity levels. While the micelles have good physicochemical properties, adjustments are needed to enhance biocompatibility for potential in vivo applications. The proof-of-principle in vivo^19^F-MR imaging of non-toxic NPs demonstrated a sufficient signal, strengthening their potential utility for future preclinical applications.

## Electronic supplementary material

Below is the link to the electronic supplementary material.

Supplementary Material 1

Supplementary Material 2

## Data Availability

The datasets used and analysed during the current study available from the corresponding author on reasonable request.
