# Supplementary material for: Cationic fluorinated micelles for cell labeling and 19F-MR imaging
Source: Sci Rep. 2024 Sep 30;14:22613. doi: 10.1038/s41598-024-73511-8 (PMC11442823; doi:10.1038/s41598-024-73511-8)
Supplement: Supplementary file 1 — Supplementary Material 1 [file 41598_2024_73511_MOESM1_ESM.docx]

**Supplementary Information**

**Table S1.** Properties of statistical copolymers of HEAM and APTMA that were used as macroCTAs in the block copolymer nanoparticle synthesis.

| Polymer | CTA^1^ | End-group^2^ | *F*_APTMA_ (%)^3^ | *M*_n_ (kDa)^4^ | *Ð*^4^ |
| --- | --- | --- | --- | --- | --- |
| B1 | MBTP | -COOMe | 0 | 23.5^5^ | 1.37^5^ |
| B2 | MBTP | -COOMe | 10 | 13.3 | 1.37 |
| B3 | MBTP | -COOMe | 20 | 18.3 | 1.36 |
| B4 | MBTP | -COOMe | 100 | 22 | 1.28 |
| BA1 | BTPA | -COO^-^ | 0 | 29.6^5^ | 1.45^5^ |
| BA2 | BTPA | -COO^-^ | 20 | 27.6 | 1.40 |
| BA3 | BTPA | -COO^-^ | 100 | n.d. | n.d. |

^1^Low molar mass chain transfer agent. ^2^Chain-end group at physiological pH. ^3^APTMA molar content in copolymer as determined by ^1^H NMR. ^4^Determined by SEC in a methanolic buffer. ^5^Determined by SEC in DMAc. n.d. = not determined.


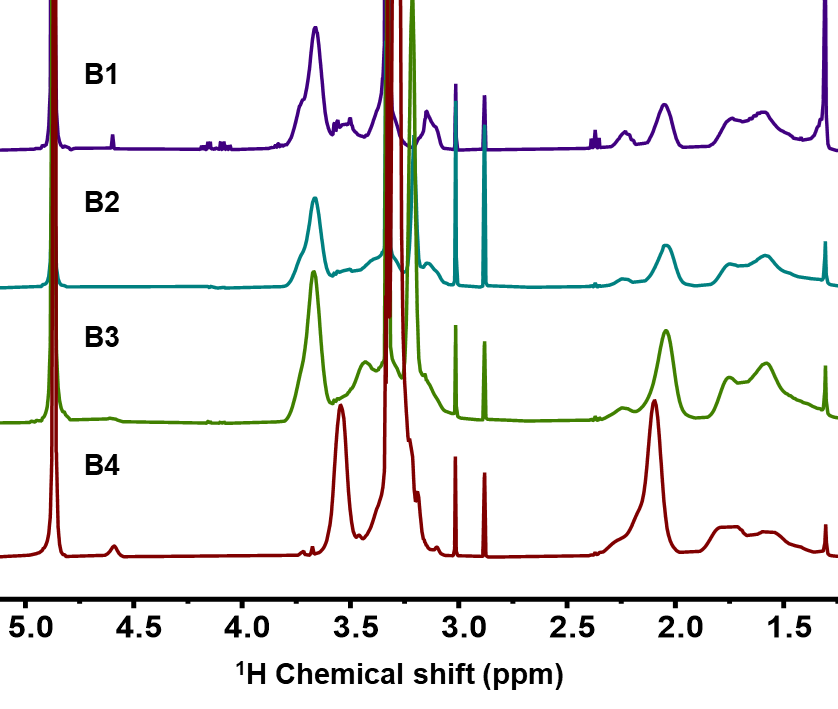


**Figure S1.** ^1^H-NMR spectra (400 MHz) of HEAM-based macro-CTAs B1-4 with controlled positive charge density in CD_3_OD.


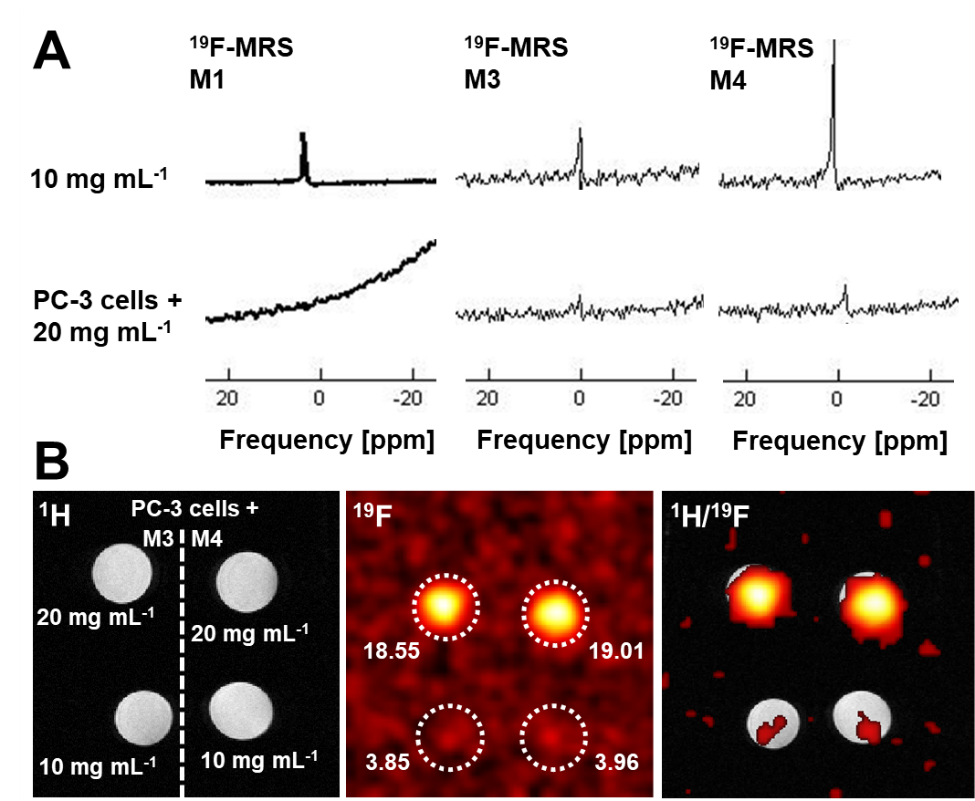


**Figure S2.** Results of the (A) ^19^F-MRS and (B) ^19^F-MRSI measurements, where M1, M3 and M4 micelles were tested for sensitivity. For the spectroscopic measurements, all initial micelles were tested in *c_pol_* = 10 mg mL^-1^ phantom and *c_pol_* = 20 mg mL^-1^ was used for cell labeling. The scan time was 33 min (M1) and 1 min (M3 – M4) and obtained using a solenoid coil. In MRSI, all probes were measured at the same time (surface coil, scan time = 1 hour). The overlaid ^1^H/^19^F-MRI is presented with the fluorine signal artificially marked in red.
