# Supplementary material for: Cationic fluorinated micelles for cell labeling and 19F-MR imaging
Source: Sci Rep. 2024 Sep 30;14:22613. doi: 10.1038/s41598-024-73511-8 (PMC11442823; doi:10.1038/s41598-024-73511-8)
Supplement: Supplementary file 2 — Supplementary Material 2 [file 41598_2024_73511_MOESM2_ESM.docx]

Cationic Fluorinated Micelles Optimized for Cell Labeling and ^19^F-MR Imaging

**Natalia Jirát-Ziółkowska, Vyshakh Manayath Panakkal, Klára Jiráková, Dominik Havlíček, Ondřej Sedláček, Daniel Jirák**

**Supplementary Information**

**Table S1.** Properties of statistical copolymers of HEAM and APTMA that were used as macroCTAs in the block copolymer nanoparticle synthesis.

| Polymer | CTA^1^ | End-group^2^ | *F*_APTMA_ (%)^3^ | *M*_n_ (kDa)^4^ | *Ð*^4^ |
| --- | --- | --- | --- | --- | --- |
| B1 | MBTP | -COOMe | 0 | 23.5^5^ | 1.37^5^ |
| B2 | MBTP | -COOMe | 10 | 13.3 | 1.37 |
| B3 | MBTP | -COOMe | 20 | 18.3 | 1.36 |
| B4 | MBTP | -COOMe | 100 | 22 | 1.28 |
| BA1 | BTPA | -COO^-^ | 0 | 29.6^5^ | 1.45^5^ |
| BA2 | BTPA | -COO^-^ | 20 | 27.6 | 1.40 |
| BA3 | BTPA | -COO^-^ | 100 | n.d. | n.d. |

^1^Low molar mass chain transfer agent. ^2^Chain-end group at physiological pH. ^3^APTMA molar content in copolymer as determined by ^1^H NMR. ^4^Determined by SEC in a methanolic buffer. ^5^Determined by SEC in DMAc. n.d. = not determined.


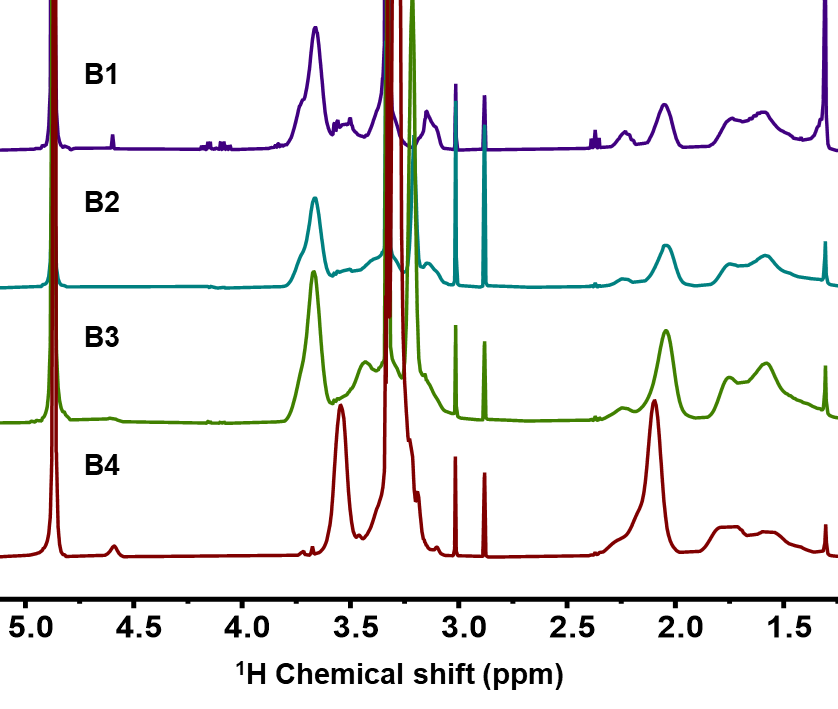


**Figure S1.** ^1^H-NMR spectra (400 MHz) of HEAM-based macro-CTAs B1-4 with controlled positive charge density in CD_3_OD.

**
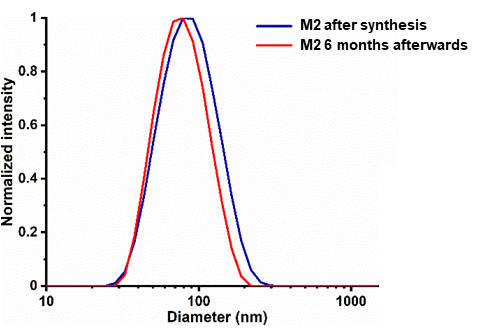
**

**Figure S2.** Hydrodynamic size distribution in water measured by DLS (*c_pol_* = 1 mg mL^-1^). DLS was performed right after the synthesis of M2 nanoparticles and six months afterwards.


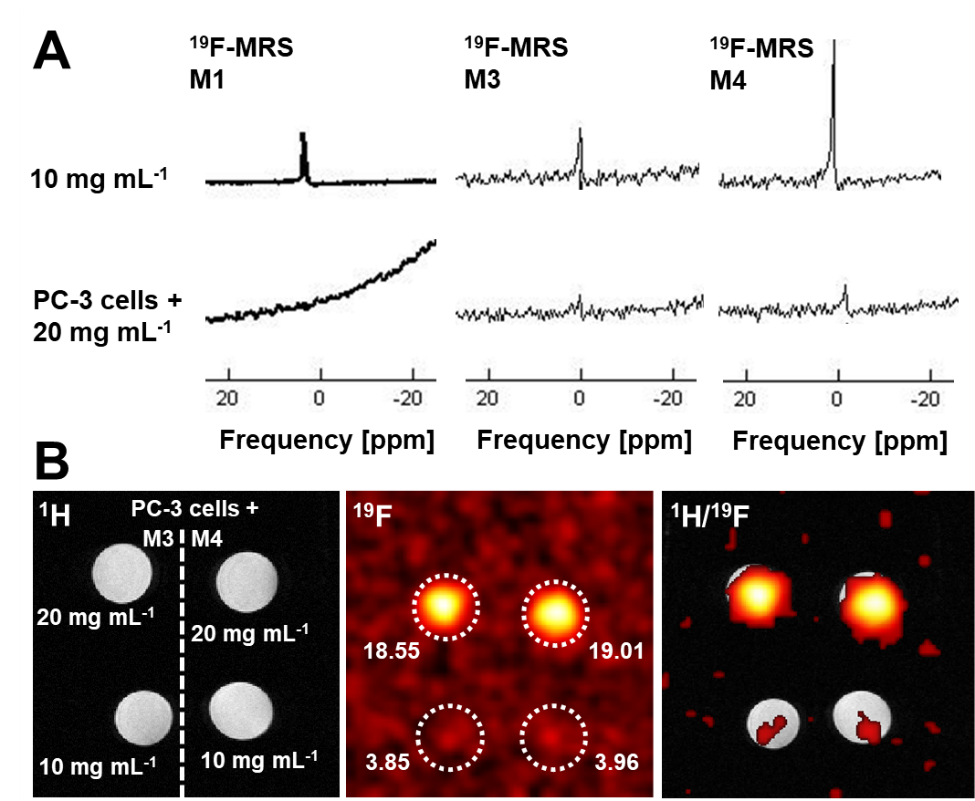


**Figure S3.** ^19^F-MRS/MRSI of micelles. (A) ^19^F-MRS and (B) ^19^F-MRSI measurements, where M1, M3 and M4 micelles were tested for sensitivity. For the spectroscopic measurements, all initial micelles were tested in phantom (*c_pol_* = 10 mg mL^-1^). For cell labeling, a concentration of *c_pol_* = 20 mg mL^-1^ was used. The scan time was 33 min for M1 and 1 min for M3 and M4, with measurements obtained using a solenoid coil. In MRSI, all probes were measured simultaneously using a surface coil with a scan time of 1 hour. The overlaid ^1^H/^19^F-MRI is presented with the fluorine signal artificially marked in red.


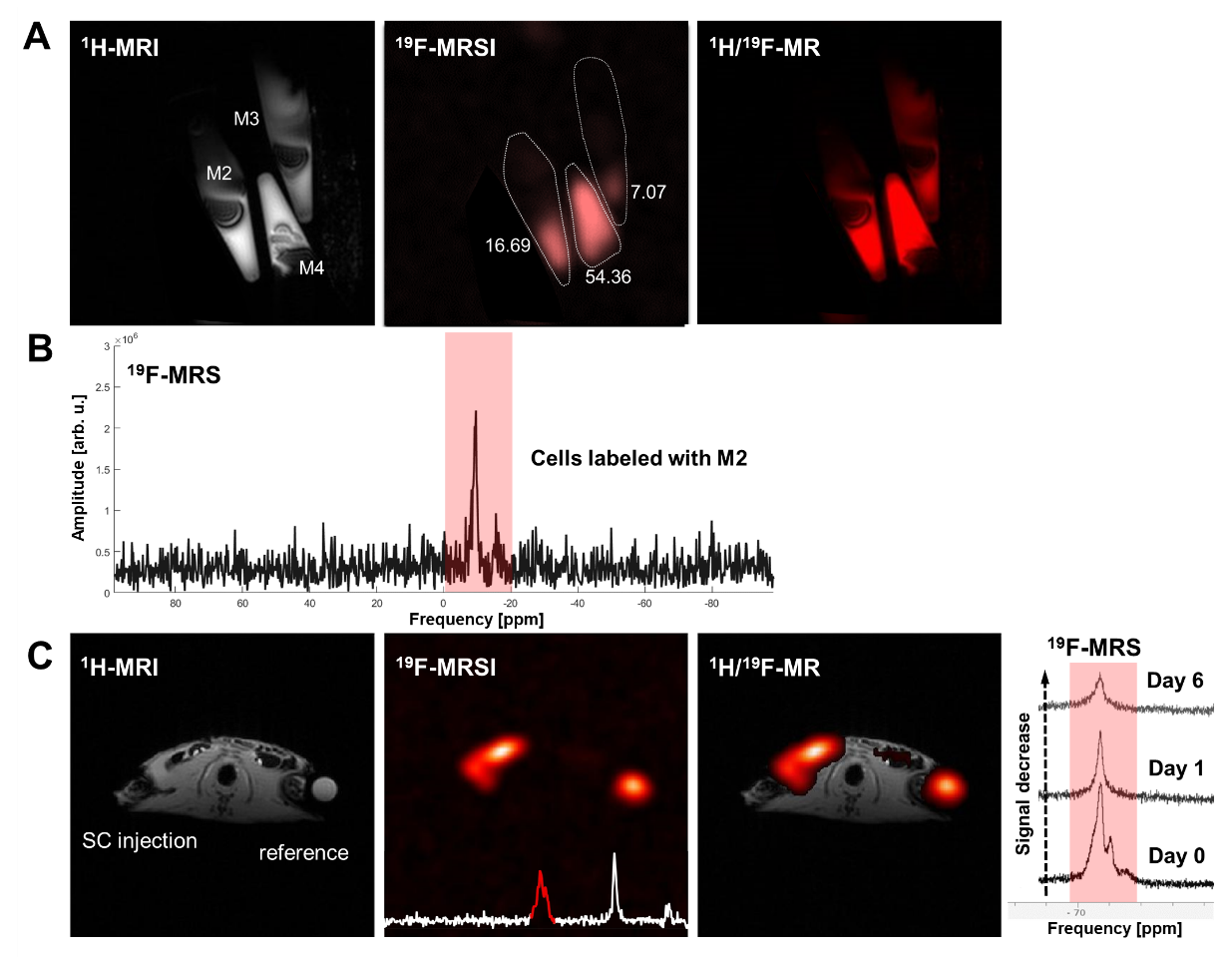


**Figure S4**. A proof-of-principle detection of micelles by ^1^H/^19^F-MR on a 7 T scanner. (A) M2, M3 and M4 micelles (*c_pol_* = 60 mg mL^-1^) with resulting SNR on ^19^F-MRSI. (B) 4T1 cells labeled with M2 (*c_pol_* = 20 mg mL^-1^) measured using ^19^F-MRS. (C) *In vivo* MR of a healthy mouse with subcutaneously administered M2 nanoparticles (Day 0, *c_pol_* = 60 mg mL^-1^) and a reference on the opposite side measured over time (Day 0 – Day 6). Anatomical ^1^H-MRI image (left), ^19^F-MRS/MRSI (middle), overlaid ^19^F- and ^1^H-MR image (right). All measurements were obtained using a surface coil. The overlaid ^1^H/^19^F-MR signal is presented with the fluorine signal artificially marked in red.
